# Validity of Chatbot Use for Mental Health Assessment: Experimental Study

**DOI:** 10.2196/28082

**Published:** 2022-10-31

**Authors:** Anita Schick, Jasper Feine, Stefan Morana, Alexander Maedche, Ulrich Reininghaus

**Affiliations:** 1 Department of Public Mental Health, Central Institute of Mental Health Medical Faculty Mannheim Heidelberg University Mannheim Germany; 2 Institute of Information Systems and Marketing Karlsruhe Institute of Technology Karlsruhe Germany; 3 Junior Professorship for Digital Transformation and Information Systems Saarland University Saarbruecken Germany

**Keywords:** chatbot, distress, monitoring, mobile health, social desirability, social presence

## Abstract

**Background:**

Mental disorders in adolescence and young adulthood are major public health concerns. Digital tools such as text-based conversational agents (ie, chatbots) are a promising technology for facilitating mental health assessment. However, the human-like interaction style of chatbots may induce potential biases, such as socially desirable responding (SDR), and may require further effort to complete assessments.

**Objective:**

This study aimed to investigate the convergent and discriminant validity of chatbots for mental health assessments, the effect of assessment mode on SDR, and the effort required by participants for assessments using chatbots compared with established modes.

**Methods:**

In a counterbalanced within-subject design, we assessed 2 different constructs—psychological distress (Kessler Psychological Distress Scale and Brief Symptom Inventory-18) and problematic alcohol use (Alcohol Use Disorders Identification Test-3)—in 3 modes (chatbot, paper-and-pencil, and web-based), and examined convergent and discriminant validity. In addition, we investigated the effect of mode on SDR, controlling for perceived sensitivity of items and individuals’ tendency to respond in a socially desirable way, and we also assessed the perceived social presence of modes. Including a between-subject condition, we further investigated whether SDR is increased in chatbot assessments when applied in a self-report setting versus when human interaction may be expected. Finally, the effort (ie, complexity, difficulty, burden, and time) required to complete the assessments was investigated.

**Results:**

A total of 146 young adults (mean age 24, SD 6.42 years; n=67, 45.9% female) were recruited from a research panel for laboratory experiments. The results revealed high positive correlations (all *P*<.001) of measures of the same construct across different modes, indicating the convergent validity of chatbot assessments. Furthermore, there were no correlations between the distinct constructs, indicating discriminant validity. Moreover, there were no differences in SDR between modes and whether human interaction was expected, although the perceived social presence of the chatbot mode was higher than that of the established modes (*P*<.001). Finally, greater effort (all *P*<.05) and more time were needed to complete chatbot assessments than for completing the established modes (*P*<.001).

**Conclusions:**

Our findings suggest that chatbots may yield valid results. Furthermore, an understanding of chatbot design trade-offs in terms of potential strengths (ie, increased social presence) and limitations (ie, increased effort) when assessing mental health were established.

## Introduction

### Background

Mental disorders are a leading cause of disease burden in high-income countries and first emerge in adolescence and young adulthood [1]. Thus, mental health in young people is a major public health concern [2]. However, psychological help remains difficult to access [3]. To address this problem, digital technologies provide a scalable alternative for accessing low-threshold psychological assessments, digital diagnostics, and interventions [4]. In particular, digital technologies can support the early detection of symptoms, diagnostics, and treatment as they may improve access to mental health services for difficult-to-reach populations without requiring on-site visits using desktop PCs, tablets, or mobile devices [5].

Text-based conversational agents (ie, chatbots) are a promising digital technology in this context [6-12]. Chatbots interact with users via natural language [13], keeping individuals engaged in the task at hand, thereby increasing adherence [10,14]. Chatbots as software-based systems enabling asynchronous interactions have received increasing attention during the COVID-19 pandemic to provide information about infection numbers, rules, and restrictions [15], thereby improving health literacy and reducing the burden on the health care system. In addition, chatbots have been investigated in several studies and applied to assess or monitor mental health [16], deliver information for improving mental health literacy [9,14,15,17], and assist and compound therapy sessions as guided or blended care [18-22]. Irrespective of the popularity of chatbots, reviews of their application in the context of (mental) health emphasize the quasi-experimental nature of studies and the need to empirically evaluate their impact [7,16,23-26]. Specifically, for wider application, the extent to which a new mode for assessing a construct (eg, chatbots assessing psychological distress) converges with established assessment modes of the same construct (ie, the convergent validity) needs to be demonstrated. In addition, discriminant validity (ie, the extent to which a construct can be distinguished from another, unrelated construct) needs to be examined. However, to date, no study has specifically examined the validity of chatbot use in assessing mental health.

This is particularly relevant, as there is evidence that individuals preconsciously attribute human characteristics to chatbots because of increased perceived social presence [27-30]. Social presence can be defined as “the degree of salience of the other person in a mediated communication and the consequent salience of their interpersonal interactions” [31]. Thus, individuals may feel a sense of personal, sociable, and sensitive human contact during a computer-mediated interaction. Although an increase in perceived social presence in face-to-face interviews has been found to increase response biases [32-35], self-reported assessments associated with reduced social presence have demonstrated reliability and validity compared with, for example, face-to-face assessments [36-40]. However, the natural language interaction style of chatbots may yield response biases such as socially desirable responding (SDR) [32,41,42], where participants disclose less socially sensitive information, which might be of special interest when applying for mental health assessment.

Previous evidence indicates that SDR may increase when individuals expect their responses to be immediately reviewed and evaluated by a researcher [33,43,44]. If chatbots are perceived as human actors [42,45], this may lead individuals to believe that their responses are immediately reviewed and evaluated. This may bias the results compared with web-based assessments that are not presented with a natural language interface and would limit the application of chatbots in remote settings, in which information is not immediately shared with a clinician. Consequently, it is necessary to investigate whether SDR is increased in settings where individuals do or do not expect their responses to be immediately reviewed when assessed by chatbots.

Finally, there is evidence that chatbots may not necessarily reduce participants’ efforts to complete the assessments [46,47]. Although the completion of assessments delivered via established assessment modes is simple (eg, by ticking a box or clicking a button), chatbots require more complex natural language interactions. This may increase the cognitive resources and duration required for assessments using chatbots [46,47]. Thus, it is necessary to investigate whether individuals using a chatbot perceive assessments as more effortful (ie, as being more complex, difficult, and associated with more burden), as well as whether they require more time to complete assessments than when using established modes.

### Objectives

This study aimed to investigate (1) the convergent and discriminant validity of assessments using chatbots, (2) the effect of assessments using chatbots on SDR, and (3) the effort of assessments using chatbots compared with established paper-and-pencil and web-based assessment modes. Specifically, we proposed the following hypotheses: chatbots applied to assess mental health (ie, psychological distress and problematic alcohol use) in healthy young adults will show high convergent validity with established assessment modes and high discriminant validity (hypothesis 1); increase SDR compared with established assessment modes (hypothesis 2a); increase SDR compared with established modes, especially in settings where individuals do not expect their responses to be immediately reviewed by the research team (hypothesis 2b); and be perceived as more effortful (ie, complex, difficult, and associated with more burden) and will require more time to complete than established assessment modes (hypothesis 3).

## Methods

### Experimental Design

A laboratory experiment applying a randomized mixed design with 3 within-subject conditions and 2 between-subject conditions was conducted. The within-subject manipulation comprised three assessment modes: (1) paper-and-pencil mode, (2) desktop computer using a typical web-based screening mode (web-based), and (3) assessment on a desktop computer screen using a chatbot (chatbot). For the between-subject manipulation, we randomly assigned participants to two conditions: participants in condition A (low-stake condition) were informed that their responses were not immediately reviewed by the research team, and participants in condition B (high-stake condition) were informed that their responses were immediately reviewed and may require a follow-up interaction with the research team.

### Procedure and Manipulation

**Figure 1:**
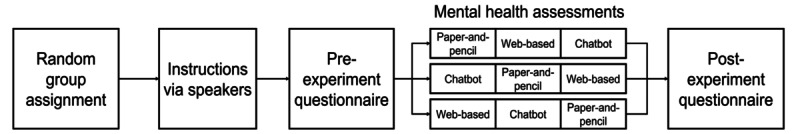
Experimental procedure.

The experimental procedure is illustrated in Figure 1. First, participants were assigned to 1 of the 2 conditions. We conducted 6 experimental sessions on 2 consecutive days, with 3 sessions assigned to condition A (low-stake condition) and 3 sessions assigned to condition B (high-stake condition). After signing the informed consent form, participants were seated in front of a desktop computer screen in single air-conditioned and soundproof test chambers. Second, participants listened to a prerecorded voice message explaining the experimental procedure and the instructions. Participants in condition B were informed of their individual participation numbers. The number was displayed on the computer screen throughout the experiment: in the web-based mode, LimeSurvey [48] displayed the participant number at the top of the screen; in the paper-and-pencil mode, participants had to write their participant number on the questionnaire; and in the chatbot mode, participants were addressed with their participant number (ie, “Hello participant 324352”) displayed in the chat window below their responses.

**Figure 2:**
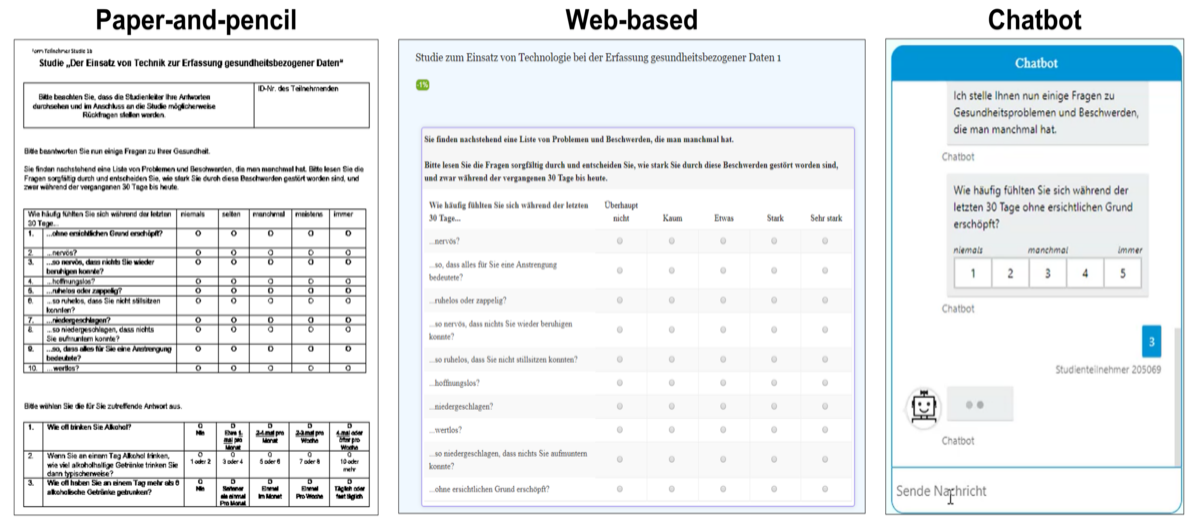
Investigated assessment modes (displayed in German).

Next, the computer screen was automatically turned on, and the experiment began with a pre-experiment questionnaire using LimeSurvey [48]. Subsequently, mental health was assessed using the 3 different modes in a counterbalanced order (Figure 2). The web-based mode used the default LimeSurvey question format. The paper-and-pencil mode comprised a printout of the digital version, which was placed in an envelope in each chamber. After completing the paper-and-pencil mode, the participants were asked to place the questionnaire in the envelope and seal the envelope with adhesive tape. The chatbot mode was developed using the Microsoft Bot Framework [49] and was integrated into LimeSurvey. The chatbot presented the items one after another and offered 2 ways of responding, either by natural language or by selecting a value (implemented as a button). The chatbot incorporated the following social cues to further increase perceived social presence [28,30]: an anthropomorphic icon [50], the capability to engage in small talk [51], a dynamically calculated response delay based on the length of the response [30], and a typing indicator (3 moving dots indicating that a message is being prepared) [52]. Microsoft’s personality chat small talk package was used to enable a small talk interaction. This knowledge base was implemented in Microsoft’s QnA Maker and was connected to the chatbot. When the QnA model identified a high match with an incoming user message, the chatbot answered with an appropriate small talk phrase. However, the chatbot’s capabilities were restricted, and no sophisticated conversations were possible. For example, the small talk included greetings such as “Hi/Hello/Good Morning!” and “How are you?”; however, the small talk did not account for the context. After answering with a small talk phrase, the chatbot always repeated the prior question. In addition, we did not record the log files of the chats. On the continuum of machine-like to human-like appearance, we chose an intermediate design to avoid the induction of negative affect toward the chatbot, which has been postulated for the increased human-likeness of robots according to the uncanny valley theory by Mori [53]. In addition, we chose the name indicator *Chatbot*, as robotic names have been reported to be positively perceived [6].

Finally, the participants answered a postexperiment questionnaire using LimeSurvey. They were then debriefed and received their compensation.

### Measures

In the pre-experiment questionnaire, we assessed demographic variables (eg, sex, age, and education), followed by questions on participants’ prior experience with using specific technologies (ie, internet and chatbots) with regard to health questions. Next, their experience with paper-and-pencil and web-based surveys, as well as with chatbots, was assessed on a scale ranging from 1 (no experience) to 5 (very much experience).

#### Balanced Inventory of Desirable Responding

On the one hand, we applied the short form of the Balanced Inventory of Desirable Responding (BIDR) scale, which comprises two subscales: self-deceptive enhancement and impression management [54,55] to capture SDR. The 18 items were rated on a 7-point Likert scale ranging from 1 (strongly disagree) to 7 (strongly agree). We calculated the total score for each scale and the BIDR total score, which ranged from 1 to 126.

On the other hand, we operationalized SDR as a response shift; that is, a change in participant’s mental health scores between repeated assessments in different modes.

#### Mental Health Measures

Mental health was assessed using the following measures in all 3 modes.

##### Kessler Psychological Distress Scale

Psychological distress in the past month was measured using the Kessler Psychological Distress Scale (K10) [56]. This 10-item self-report questionnaire is rated on a Likert scale ranging from 1 (never) to 5 (always). The K10 total score was calculated. Strong psychometric properties of the K10 have been reported [56].

##### Brief Symptom Inventory

We used the short form of the Brief Symptom Inventory (BSI-18) [57,58] to assess psychological distress in the past 7 days. Participants indicated whether they had experienced 18 symptoms, comprising 3 dimensions: somatization, depression, and anxiety. The items were rated on a scale from 1 (not at all) to 5 (very much). We calculated the total score indicating general distress (BSI–General Severity Index) [58].

##### Alcohol Use Disorders Identification Test-3

We assessed alcohol use by applying the Alcohol Use Disorders Identification Test (AUDIT)–3 questionnaire [59,60], which has been shown to perform similarly well as the AUDIT-10 in detecting risky drinking behavior [60]. The items were presented on a 5-point scale with different labels asking about the amount of alcohol consumption. The total AUDIT-3 score was calculated.

The time at the beginning and end of data collection in each mode was recorded. In the postexperiment questionnaire, participants had to rank the 3 modes regarding complexity, difficulty, and burden. Subsequently, we asked participants to rate others’ discomfort when answering each item of the mental health measures, thereby deriving a measure of subjective sensitivity in line with Bradburn et al [61].

### Attention and Manipulation Checks

In the attention check, participants had to select a specific item on a Likert scale to verify that they carefully followed the instructions (“Please select the answer very often”). To test the within-subject manipulation, we investigated differences in the perceived social presence of each mode using the 4 items by Gefen and Straub [62], which were rated on a 7-point Likert scale. The internal consistency of the perceived social presence of the 3 modes was high (Cronbach α>.89).

Furthermore, participants had to indicate in the postexperiment questionnaire whether their answers were immediately reviewed, in line with Fisher [44] (between-subject manipulation check).

### Power Analysis and Recruitment

An a priori analysis in G*Power software (Heinrich-Heine-Universität Düsseldorf) [63] estimated a total sample size of 116 (α=.05; *f*=0.15; Cohen *d*=0.95). For recruitment, we invited individuals registered with the university’s research panel, comprising mainly students from the Karlsruhe Institute of Technology. The experiment lasted 45 minutes on average and participants were compensated for their participation with €8 (US $8.06) after the experiment.

### Statistical Analysis

SPSS Statistics (version 25; IBM Corp) and STATA (version 16.0; StataCorp) were used to analyze the data. Participant characteristics were summarized using means and SDs for continuous variables and frequencies and percentages for dichotomous variables. To investigate differences between groups, we calculated the ANOVAs for individuals’ tendency to respond as socially desirable (BIDR) and the perceived sensitivity of each measure (K10, BSI-18, and AUDIT-3). Furthermore, differences between prior experience with, as well as the perceived social presence of, modes were investigated by calculating repeated-measures ANOVAs (rmANOVAs). As data on prior experience (*χ*^2^_2_=46.4; *P*<.001) and perceived social presence (*χ*^2^_2_=49.5; *P*<.001) violated the assumptions of sphericity, Huynh-Feldt corrections were applied.

The internal consistency of the mental health measures for each mode was evaluated using Cronbach α. Next, the test-retest reliabilities of the chatbot-based, paper-and-pencil–based, and desktop-based assessment modes were evaluated by calculating intraclass correlation coefficients (ICCs) ranging from 0 (no agreement) to 1 (perfect agreement).

To test hypothesis 1 on the discriminant and convergent validity of assessment modes, we calculated Pearson correlations and applied Bonferroni correction to account for multiple testing. In line with the multitrait-multimethod approach by Campbell and Fiske [64], we tested 3 independent assessment modes with 2 different constructs—psychological distress (K10 and BSI-18) and problematic alcohol use (AUDIT-3)—to derive discriminant and convergent validity. Validity is indicated by a correlation coefficient of ≥0.50 [63].

To test hypothesis 2a, we calculated repeated-measures analyses of covariance (rmANCOVAs) with the within-subject factor mode (paper-and-pencil, web-based, and chatbot) and the following covariates: (1) perceived sensitivity of the items and (2) individuals’ tendency to respond socially desirable (BIDR). Sex was also included as a control variable in all the analyses. Lavene test revealed the homogeneity of variances for all 3 measures. As the AUDIT-3 data violated the assumptions of sphericity (*χ*^2^_2_=13.2; *P*=.001), the Huynh-Feldt correction was applied in the rmANCOVA.

To test hypothesis 2b, rmANCOVAs with the within-subject factor mode (paper-and-pencil, web-based, and chatbot) and condition (A and B) as additional covariates were calculated. Lavene test revealed the homogeneity of variances for all modes. Again, the AUDIT-3 data violated the assumption of sphericity (*χ*^2^_2_=13.4; *P*=.001), and the Huynh-Feldt correction was applied.

To test hypothesis 3 on the effort of assessment, we analyzed the ranked-ordered data on complexity, difficulty, and burden by calculating Friedman tests and Dunn-Bonferroni post hoc signed-rank tests for pairwise comparisons. Differences in the duration to complete the assessments were investigated by calculating rmANOVAs with the within-subject factor mode (paper-and-pencil, web-based, and chatbot). As the data violated the assumptions of sphericity (*χ*^2^_2_=9.1; *P*=.01), the Huynh-Feldt correction was applied.

### Ethics Approval

The experiment took place at the Karlsruhe Decision and Design Lab, adhering to its procedural and ethical guidelines. No ethics approval was applied for as participants were recruited from the registered participant panel of healthy students. Individuals voluntarily participated after being fully informed about the study procedures and signing the informed consent form. No identifying data were collected.

## Results

### Sample Characteristics

We invited all individuals registered in the university’s research panel to participate in the experiment. A total of 155 individuals participated in the study, of whom 9 (5.8%) participants were excluded as they failed the attention check, indicating that they may not have followed the instructions of the experiment or had not read the individual items carefully. Consequently, 146 participants were included in the analysis, of whom 72 (49.3%) were in condition A and 74 (50.7%) were in condition B.

The sample characteristics and control variables are presented in Table 1. Overall, we investigated a sample of young students from which most participants had a high school or bachelor’s degree. In addition, two-thirds of the participants (100/146, 68.5%) indicated that they had used the internet to access information on mental health before. However, only 4.1% (6/146) of participants replied having interacted with a chatbot in a health-related context before. Prior experience with assessment modes differed across the 3 modes, as revealed by the rmANOVA (*F*_1.58, 229.39_=225.23; *P*<.001). Post hoc analyses with a Bonferroni adjustment further showed that the experience with chatbots (mean 1.73*,* SD 1.02) was lower than the experience with paper-and-pencil surveys (mean 3.45*,* SD 0.85), as well as the experience with web-based surveys (mean 3.52*,* SD 0.82, all *P<*.001). Experience with paper-and-pencil surveys did not significantly differ from that with web-based surveys (*P*=.78). Individuals’ tendency to respond socially desirable, as measured using the BIDR, did not differ between conditions (*F*_1,144_=0.131; *P*=.72) and was centered on the mean (*W*_146_=0.98; *P*=.09). The perceived sensitivity of the items of the 3 mental health measures did not differ between the 2 conditions (all *P*>.47) but differed between the 3 measures (*F*_1.41, 88.22_ =105.64; *P*<.001). Post hoc analyses with Bonferroni adjustment indicated that AUDIT-3 items (mean 3.39, SD 1.07) were rated as more sensitive than K10 items (mean 2.59, SD 0.66; *P<*.001), as well as BSI-18 items (mean 2.33*,* SD 2.33, *P<*.001). Furthermore, the K10 items (mean 2.59, SD 0.66) were perceived to be more sensitive than the BSI-18 items (mean 2.33*,* SD 0.58; *P<*.001).

**Table 1:** Sample characteristics (N=146).

Variable		Full sample	Low-stake condition (n=72)	High-stake condition (n=74)
Age (years), mean (SD)		24.2 (6.42)	23.44 (6.06)	24.93 (6.71)
Female, n (%)		67 (45.9)	30 (41.7)	37 (50)
**Education, n (%)**				
	Middle school	3 (2.1)	2 (2.8)	1 (1.4)
	High school	89 (60.9)	43 (59.7)	46 (62.2)
	Bachelor’s	46 (31.5)	25 (34.7)	21 (28.4)
	Master’s	8 (5.5)	2 (2.8)	6 (8.1)
**Technology experience^a^, n (%)**				
	Internet	100 (68.5)	51 (70.8)	49 (66.2)
	Chatbot	6 (4.1)	2 (2.8)	4 (5.4)
**Survey experience, mean (SD)**				
	Paper-and-pencil	3.45 (0.85)	3.53 (0.87)	3.36 (0.82)
	Web-based	3.52 (0.82)	3.57 (0.77)	3.47 (0.88)
	Chatbot	1.73 (1.02)	1.64 (0.86)	1.82 (1.15)
**Social desirability, mean (SD)**				
	BIDR^b^ total	83.60 (9.38)	83.32 (9.15)	83.89 (9.67)
	BIDR-SDE^c^	41.55 (5.00)	41.65 (4.62)	41.46 (5.39)
	BIDR-IM^d^	42.05 (6.93)	41.68 (7.06)	42.43 (6.82)
**Sensitivity of measures,** **mean (SD)**				
	K10^e^	2.59 (0.66)	2.61 (0.71)	2.57 (0.62)
	BSI-18^f^	2.33 (0.58)	2.34 (0.58)	2.33 (0.57)
	AUDIT-3^g^	3.39 (1.07)	3.45 (1.07)	3.32 (1.08)

^a^Number of participants who previously used technology in a health-related context.

^b^BIDR: Balanced Inventory of Desirable Responding.

^c^BIDR-SDE: Balanced Inventory of Desirable Responding–Self-deceptive enhancement.

^d^BIDR-IM: Balanced Inventory of Desirable Responding–Impression management.

^e^K10: Kessler Psychological Distress Scale.

^f^BSI-18: Brief Symptom Inventory-18.

^g^AUDIT-3: Alcohol Use Disorders Identification Test-3.

### Manipulation Checks

With regard to the within-subject manipulation, the results of the rmANOVA revealed a significant effect of mode on perceived social presence (*F*_1.56_*_,_*_226.67_=61.96; *P<*.001), with social presence rated highest in the chatbot mode (mean 2.74, SD=1.51) compared with the web-based mode (mean 1.48*,* SD 0.88; *P<*.001) and paper-and-pencil mode (mean 1.79, SD 1.21; *P<*.001).

Responses to the between-subject manipulation check showed that 93.2% (136/146) of participants provided a correct answer—2.7% (4/146) of individuals with wrong answers were in condition A and 4.1% (6/146) were in condition B—and were aware of their condition. Consequently, we concluded that both within-subject and between-subject manipulations were successful.

### Reliability of Chatbots for Mental Health Assessments

Table 2 displays the mean, SD, Cronbach α, and ICC for the mental health measures in each mode by condition. The ICCs of the paper-based, desktop-based, and chatbot modes were high and ranged between 0.96 and 1.00, indicating excellent agreement across modes and a high test-retest reliability. Cronbach α did not strongly vary between modes and ranged between 0.74 and 0.92, indicating an acceptable to excellent internal consistency of the measures.

**Table 2:** Internal consistency and test-retest reliability of mental health assessments.

Measure and mode			Full sample				Low-stake condition					High-stake condition					ICC^a^	
			Values, mean (SD)		Cronbach α		Values, mean (SD)		Cronbach α		Values, mean (SD)			Cronbach α				
**K10^b^**																		0.96
	Paper-based	19.36 (6.53)		.89		19.44 (5.66)		.84		19.28 (7.31)			.92					
	Web-based	19.77 (6.67)		.88		19.47 (5.63)		.82		20.05 (7.57)			.91					
	Chatbot-based	19.7 (6.45)		.86		19.43 (5.81)		.82		19.95 (7.04)			.89					
**BSI-18^c^**																		0.99
	Paper-based	11.54 (8.45)		.86		11.35 (6.72)		.78		11.73 (9.9)			.9					
	Web-based	11.56 (8.89)		.87		11.29 (7.48)		.82		11.81 (10.12)			90					
	Chatbot-based	11.09 (8.4)		.86		10.71 (7.09)		.8		11.46 (9.54)			.89					
**AUDIT-3^d^**																		1.00
	Paper-based	3.42 (2.45)		.80		3.50 (2.60)		.85		3.34 (2.30)			.74					
	Web-based	3.40 (2.44)		.81		3.49 (2.62)		.86		3.32 (2.28)			.75					
	Chatbot-based	3.43 (2.49)		.82		3.49 (2.64)		.86		3.38 (2.36)			.76					

^a^ICC: intraclass correlation coefficient.

^b^K10: Kessler Psychological Distress Scale.

^c^BSI-18: Brief Symptom Inventory-18.

^d^AUDIT-3: Alcohol Use Disorders Identification Test-3.

### Validity of Assessments Using Chatbots (Hypothesis 1)

As depicted in Table 3, there were strong positive correlations between the measures of psychological distress (K10 and BSI-18) assessed by the different modes, with correlation coefficients ranging from 0.83 to 0.96, indicating convergent validity. Furthermore, there were strong positive correlations between the AUDIT-3 scores assessed using the different modes. There were no significant correlations among AUDIT-3, K10, and BSI-18 after Bonferroni correction, indicating discriminant validity between the different constructs.

**Table 3:** Pearson correlation of questionnaires and modes. Higher numbers reflect a stronger association between variables.

Mode		K10^a^				BSI-18^b^				AUDIT-3^c^		
		Paper-based *r* (*P* value^d^)	Web-based *r* (*P* value)	Chatbot-based *r* (*P* value)	Paper-based *r* (*P* value)		Web-based *r* (*P* value)	Chatbot-based *r* (*P* value)	Paper-based *r* (*P* value)		Web-based *r* (*P* value)	Chatbot-based *r* (*P* value)
**K10**												
	Paper-based	1	0.89 (<.001)	0.88 (<.001)	0.89 (<.001)		0.83 (<.001)	0.85 (<.001)	−0.1 (.21)		−0.12 (.14)	−0.13 (.12)
	Web-based	0.89 (<.001)	1	0.87 (<.001)	0.88 (<.001)		0.89 (<.001)	0.86 (<.001)	−0.18 (.04)		−0.19 (.02)	−0.20 (.02)
	Chatbot-based	0.88 (<.001)	0.87 (<.001)	1	0.85 (<.001)		0.84 (<.001)	0.85 (<.001)	−0.09 (.27)		−0.11 (.17)	−0.12 (.16)
**BSI-18**												
	Paper-based	0.89 (<.001)	0.88 (<.001)	0.85 (<.001)	1		0.96 (<.001)	0.96 (<.001)	−0.1 (.22)		−0.12 (.15)	−0.14 (.10)
	Web-based	0.83 (<.001)	0.89 (<.001)	0.84 (<.001)	0.96 (<.001)		1	0.96 (<.001)	−0.14 (.09)		−0.16 (.06)	−0.18 (.04)
	Chatbot-based	0.85 (<.001)	0.86 (<.001)	0.85 (<.001)	0.96 (<.001)		0.96 (<.001)	1	−0.15 (.07)		−0.16 (.05)	−0.17 (.04)
**AUDIT-3**												
	Paper-based	−0.1 (.21)	−0.18 (.04)	−0.09 (.27)	−0.1 (.22)		−0.14 (.09)	−0.15 (.07)	1		0.99 (<.001)	0.99 (<.001)
	Web-based	−0.12 (.14)	−0.19 (.02)	−0.11 (.17)	−0.12 (.15)		−0.16 (.06)	−0.16 (.05)	0.99 (<.001)		1	0.99 (<.001)
	Chatbot-based	−0.13 (.12)	−0.20 (.02)	−0.12 (.16)	−0.14 (.10)		−0.18 (.04)	−0.17 (.04)	0.99 (<.001)		0.99 (<.001)	1

^a^K10: Kessler Psychological Distress Scale.

^b^BSI-18: Brief Symptom Inventory-18.

^c^AUDIT-3: Alcohol Use Disorders Identification Test-3.

^d^Unadjusted *P* value; the Bonferroni corrected significance level was computed by dividing the unadjusted *P* value by the total number of tests; that is, *P*=.05/45=.0011.

### SDR to Chatbots in Mental Health Assessments (Hypotheses 2a and 2b)

Addressing hypothesis 2a, the rmANCOVA on the effect of mode on mental health assessment revealed no main effect of mode on K10 (*F*_2,284_=0.35; *P*=.71). Moreover, there was no interaction between mode and social desirability (*F*_2,284_=0.80; *P*=.45) or perceived sensitivity of the items (*F*_2,284_=0.43; *P*=.65); however, there was a significant interaction with sex (*F*_2,284_=3.21; *P*=.04). The second mental distress measure, the BSI-18, showed similar results. The rmANCOVA revealed no significant main effect of mode on general distress (*F*_2,248_=0.90; *P*=.41). Again, there was no interaction between mode and social desirability (*F*_2,__284_=1.7; *P*=.19), sensitivity (*F*_2,284_=0.23; *P*=.80), or sex (*F*_2,284_=2.66; *P*=.07). Similarly, the rmANCOVA on AUDIT-3 scores revealed no significant main effect of mode (*F*_1_*_._*_90_*_,_*_269.57_=0.00; *P*=1.00), as well as no interaction of mode with social desirability (*F*_1.90_*_,_*_269.57_=0.01; *P*=.99), perceived sensitivity of items (*F*_1_*_._*_90_*_,_*_269.57_=0.24; *P*=.77), or sex (*F*_1.90_*_,_*_269.57_=0.33; *P*=.71).

The effect of the condition on mental health assessment (hypothesis 2b) was investigated using a second set of rmANCOVAs. The results revealed no significant interaction effect between mode and condition on psychological distress assessed by K10 (*F*_2,282_=0.91; *P*=.41), general distress assessed using the BSI (*F*_2,282_=0.29; *P*=.75), or alcohol use assessed by AUDIT-3 (*F*_1.91, 269.14_=0.55; *P*=.57).

### Difficulty of Assessments Using Chatbots (Hypothesis 3)

Table 4 shows the mean rating of complexity, difficulty, and burden. A Friedman test revealed a significant difference between the difficulty associated with the modes (*χ*^2^_2_=13.5; *P*=.001). Dunn-Bonferroni post hoc tests showed that the assessment by a chatbot was rated as significantly more difficult than using the paper-and-pencil mode (*z*=3.63; *P*=.001). Furthermore, there was a statistically significant difference in perceived complexity depending on the mode (*χ*^2^_2_=10.15; *P*=.006). Again, Dunn-Bonferroni post hoc tests showed that the chatbot assessment was ranked as more complex than the paper-and-pencil assessment (*z*=3.16; *P*=.005). In terms of burden, a Friedman test indicated that there was a statistically significant difference (*χ*^2^_2_=12.4; *P*=.002), and Dunn-Bonferroni post hoc tests further revealed that the web-based assessment required significantly less effort than the chatbot (*z*=2.64; *P*=.03) and the paper-and-pencil assessment (*z*=−3.34; *P*=.003). The analysis of duration revealed a significant effect of mode (*F*_1.91, 276.68_=186.60; *P<*.001). Post hoc analyses with Bonferroni adjustment revealed that the pairwise differences between all modes were significant (*P<*.001). The longest duration was logged to complete the chatbot assessment and the shortest duration was required to complete the web-based assessment.

**Table 4:** Effort of assessment modes.

Effort variable and mode		Rank, mean (SD)
**Complexity**		
	Paper-and-pencil	1.80 (0.84)
	Web-based	2.03 (0.66)
	Chatbot	2.17 (0.89)
**Difficulty**		
	Paper-and-pencil	1.81 (0.78)
	Web-based	1.96 (0.7)
	Chatbot	2.23 (0.9)
**Burden**		
	Paper-and-pencil	2.16 (0.79)
	Web-based	1.77 (0.73)
	Chatbot	2.08 (0.87)
**Duration (seconds)**		
	Paper-and-pencil	184.62 (79.28)
	Web-based	128.78 (56.07)
	Chatbot	265.1 (65.82)

## Discussion

### Principal Findings

This study examined the validity, effect on SDR, and effort required for the completion of chatbot-based assessments of mental health. The results revealed that all assessments of mental health (K10, BSI, and AUDIT) in each mode showed acceptable to excellent internal consistency and high test-retest reliability. High positive correlations between the measures of the same construct across different assessment modes indicated the convergent validity of the chatbot mode, and the absence of correlations between distinct constructs indicated discriminant validity (hypothesis 1). Although assessment modes were not affected by social desirability (hypothesis 2a), chatbot assessment was higher for perceived social presence. There was no evidence of an interaction between condition and mode, indicating that social desirability did not increase because of expectations around immediate follow-up contact with a researcher in the chatbot assessment mode (hypothesis 2b). Finally, in terms of participants’ effort (hypothesis 3), the assessment using a chatbot was found to be more complex, difficult, and associated with more burden than the established modes, resulting in a longer duration to complete.

### Limitations

The present findings must be considered in light of several limitations. First, the selection of a student sample may have resulted in the low external validity of the laboratory experiment. According to previous mental health assessments in the general population, our sample showed only moderate distress [65]. There is evidence that individuals disclose more information on sensitive topics such as health risk behavior in clinical settings [66]. Future research should further investigate the application of chatbots in clinical samples, as the present findings on social desirability or perceived social presence of chatbots do not readily generalize to clinical populations.

Second, we reduced the effect of between-person differences by selecting a within-person design, which had several limitations. Each participant completed questionnaires in all 3 modes, with an average break between modes of approximately 1 minute. During the break, participants rated their social presence and read the instructions in the next experimental section. The break may have been too short to minimize memory effects. In addition, all measures used Likert scales, which may have increased memory effects because of their simplicity. To address this limitation, we completely counterbalanced the order of the 3 modes in the experimental procedure. Furthermore, in a sensitivity analysis using data from only the first mode presented to the participants, we did not find any differences, which further supports the reported results (Multimedia Appendix 1, Table S1). However, other factors such as the need for consistent responses may have overcome social desirability. Again, a longer break between assessments or a between-subject design could be applied in future experiments.

Third, the lack of an effect of mode on change in mental health scores may have been a result of the experimental design or chatbot design. As mentioned previously, we did not assess social pressure; however, individuals showed stronger SDR in high-stakes assessment situations. Thus, the assessment of social pressure is recommended for future studies. Furthermore, in this experiment, the chatbot followed a procedural dialog flow using Likert scales and, in addition to basic small talk capabilities using several social cues [30], was unable to answer questions about topics other than the assessments. Although we demonstrated a higher perceived social presence of the chatbot, this may not have been sufficient to resemble the communication flow of a human interviewer. In addition, the perceived social presence of the chatbot may have led to increased expectations of participants in terms of the chatbot’s interactivity and natural language capabilities [28]. Thus, the chatbot may have raised expectations that may not have been met [67]. Consequently, future research should investigate different chatbot designs that support less restricted non–goal-oriented natural language interactions. In this regard, further experiments should evaluate the influence of social and empathic responses on mental health assessments.

Fourth, this study investigated the convergent and discriminant validity of measures and modes to assess the constructs of psychological distress and alcohol use. We aimed to reduce the participant burden by selecting only 3 measures of mental health. However, other even less related constructs could have been investigated to facilitate the evaluation of discriminant validity. This issue should be addressed in future research.

Finally, the longer duration of completing the assessment using a chatbot may have resulted from participants potentially entering their responses by typing or using the menu option. In this study, we did not assess the method of entering data that was used. In future research, either one response option should be favored or the 2 response options may be compared by applying a microrandomized design.

### Comparison With Prior Work

The use of chatbots for mental health assessment is an emerging field, and robust investigations of their positive and potential negative effects are required [16]. Given that recent studies have shown the feasibility of the application of chatbots in general, particularly in relation to monitoring [15], offering information on, as well as delivering interventions for, improving mental health [62,63], there is a need for methodological research on the use of chatbots in this context [7,16,23-26]. This appears to be particularly important in cases where chatbots may be seen as social actors (ie, human interviewers) evoking social desirability. Therefore, it needs to be shown that using chatbots for assessing mental health does not result in biased outcomes.

The application of chatbots has been previously shown to affect the collected data and either reduce [68-70] or increase [42] the SDR compared with assessments by human interviewers. Other studies have found that chatbot assessments may result in comparable results with established modes [8,46,71]. However, some studies have found this effect only in adult samples [72] or depending on the chatbot’s visual and linguistic design [42,73]. In this context, chatbots with high conversational abilities or a more human-like embodiment have been shown to elicit more SDR to socially sensitive questions than established modes [42,73]. However, this was not the case when a chatbot with fewer human-like conversational abilities was presented [42,73], which is consistent with findings of this study. Thus, an assessment using a chatbot with the presented design and procedural dialog flow does not seem to induce additional SDR. Despite this finding, it may be of interest to develop chatbots with high conversational abilities as these may enhance adherence and increase compliance, for example, in digital interventions [8,11,21,24]. This is particularly important for delivering interventions and building stable human-chatbot interactions [51]. Therefore, further research on chatbots is required, for example, in which different conversational interaction strategies may be applied. A promising approach may be to enable reciprocal self-disclosure, in which the chatbot reveals sensitive information, as this has been shown to result in a reciprocal effect on promoting individuals’ self-disclosure [70], as well as perceived intimacy and enjoyment [74]. Another promising approach may be the application of contingent interaction strategies, as individuals disclose more information on a website if contingent questions depending on previous interactions are displayed [75]. Moreover, voice-based conversational agents may improve response quality to sensitive questions [76]. However, more research on the design of voice-based conversational agents for mental health assessment is required [77]. In addition, unconstrained natural language input to conversational agents poses safety risks that must be evaluated thoroughly. As recently shown by Bickmore et al [78], voice-based assistants failed more than half of the time when presented with medical inquiries. Therefore, further evaluation of human-computer interactions and education about the capabilities of conversational agents is required.

In contrast to previous findings on assessments using chatbots reporting higher data quality or more engagement [8,9,11,47,69], we showed that chatbot assessments were more difficult, complex, and associated with more burden to complete than assessments using established modes. In addition, more time was required to complete the assessments. The latter has been previously shown [47] and may result from the increased cognitive demand of a communication flow, where an individual must decode and aggregate the impression-bearing and relational functions conveyed in computer-mediated communication [79]. In addition, increased effort may result from individual preferences or prior experiences with chatbots in other contexts. It has been shown that populations with high health literacy rates prefer established modes because of their efficiency and ability to proceed at their own pace [46]. This may be particularly relevant in a sample of young students. Furthermore, this finding is in line with the communication literature arguing that simple tasks may be conducted more efficiently through learner media [80]. Thus, simple tasks such as selecting Likert scale items in mental health questionnaires may be more efficiently conducted through the use of established modes such as paper-and-pencil or web-based assessments [81]. This may imply that the best application area of chatbots in mental health may not be symptom monitoring or screening but rather providing information or delivering an intervention in unstructured natural language interactions. Recent evidence supports the use of chatbot-based interventions as they have been found to perform equally well as standard treatment methods (eg, face-to-face and telephone counseling) [7].

This work provides further evidence on the use of chatbots to assess mental health on site in clinics but also in asynchronous remote medical interactions (eg, at home) [17,70,82]. As the assessment modes between conditions did not differ, the results show that the application of a chatbot results in valid responses, regardless of whether the data are immediately reviewed and evaluated by a human actor [70,83]. Therefore, chatbots have the potential to reduce the workload in clinical settings by providing valid remote assessments, which is especially necessary for situations in which the medical system is at its limits. As stated by Miner et al [15], chatbots may be a digital solution that may help provide information, monitor symptoms, and even reduce psychosocial consequences during the COVID-19 pandemic. Recently, several chatbots for monitoring COVID-19 symptoms have been published, as reviewed by Golinelli et al [84]. In contrast to other mental health apps, chatbots have the advantage of providing communication that may additionally help to reduce loneliness during means of physical distancing [85,86]. For example, it has been shown that users may develop a strong social relationship with a chatbot when it expresses empathetic support [21,51,85,87-90]. Moreover, promising real-world examples of empathetic mental health chatbots have shown their effectiveness in practice, such as the mobile app chatbots Wysa [85], Woebot [6], and Replika [91]; however, they have also raised ethical concerns [10]. Thus, the application of chatbots in mental health research and practice may depend on the specific application (symptom monitoring vs guided intervention) and its potential advantages (ie, increased social presence) and disadvantages (ie, increased effort) while respecting users’ privacy and safety.

### Conclusions

These findings provide evidence of the validity of chatbots as digital technology for mental health assessment. In particular, when paper-and-pencil assessments are not applicable (eg, remote assessments in eHealth settings) or when it may be beneficial to increase perceived social presence (eg, to establish a long-term user-chatbot relationship), chatbots are promising alternatives for valid assessment of mental health without leading to socially desirable responses. However, as participants’ efforts have increased, future research on appropriate chatbot designs and interaction flow is necessary to fully leverage their advantages in compounding digital care.

## Appendix

Multimedia Appendix 1 Sensitivity analyses.

## Abbreviations

AUDIT: Alcohol Use Disorders Identification Test
BIDR: Balanced Inventory of Desirable Responding
BSI-18: Brief Symptom Inventory-18
ICC: intraclass correlation coefficient
K10: Kessler Psychological Distress Scale
rmANCOVA: repeated-measures analysis of covariance
rmANOVA: repeated-measures ANOVA
SDR: socially desirable responding
