# Supplementary material for: Validity of Chatbot Use for Mental Health Assessment: Experimental Study
Source: JMIR Mhealth Uhealth. 2022 Oct 31;10(10):e28082. doi: 10.2196/28082 (PMC9664331; doi:10.2196/28082)
Supplement: Multimedia Appendix 1 [file mhealth_v10i10e28082_app1.docx]

# Supplementary material

As a sensitivity analysis, we investigated only the first mode that was presented. Results of the first assessment of K10, BSI-18, and AUIT-3 are reported in Table S1.

Table S1. Descriptive results of the first assessment of K10, BSI-18, and AUDIT-3.

| Measure | | First Mode | | Group A + B | | Condition A | | Condition B | |
| --- | --- | --- | --- | --- | --- | --- | --- | --- | --- |
|  |  | | M^a^  (SD^b^) | | n^c^ | M  (SD) | n | M  (SD) | n |
|  |  | |  | |  |  |  |  |  |
| **K10** |  | |  | |  |  |  |  |  |
|  | P^d^ | | 2.04  (0.56) | | 44 | 2.06  (0.51) | 29 | 2.01  (0.68) | 15 |
|  | W^e^ | | 2.18  (0.72) | | 46 | 2.18  (0.60) | 22 | 2.19  (0.83) | 24 |
|  | C^f^ | | 2.07  (0.62) | | 56 | 2.10  (0.67) | 21 | 2.05  (0.59) | 35 |
| **BSI-18** |  | |  | |  |  |  |  |  |
|  | P | | 1.63  (0.35) | | 44 | 1.62  (0.34) | 29 | 1.64  (0.40) | 15 |
|  | W | | 1.70  (0.54) | | 46 | 1.76  (0.41) | 22 | 1.66  (0.64) | 24 |
|  | C | | 1.70  (0.50) | | 56 | 1.66  (0.42) | 21 | 1.72  (0.54) | 35 |
| **AUDIT-3** |  | |  | |  |  |  |  |  |
|  | P | | 2.27  (0.88) | | 44 | 2.20  (0.87) | 29 | 2.40  (0.92) | 15 |
|  | W | | 2.07  (0.87) | | 46 | 2.12  (0.95) | 22 | 2.01  (0.81) | 24 |
|  | C | | 2.13  (0.74) | | 56 | 2.22  (0.86) | 21 | 2.07  (0.67) | 35 |

^a^M= Mean, ^b^SD= Standard deviation, ^c^n = number of participants receiving the measure in the respective mode first, ^d^P= Paper-based, ^e^W= Web-based, ^f^C= Chatbot-based

Subsequently, we investigated the main effect of mode (ie, paper-based, web-based, chatbot-based) on each of the three mental health measures (ie, K10, BSI-18, AUDIT-3) and conducted several ANOVAs accounting and not accounting for condition as well as for all control variables. Results did not reveal a difference between any mode on each of the mental health measures (all p > 0.61 when accounting for condition, all p > 0.64 when not accounting for condition). This analysis provides further support for the findings of the within-subject analysis, ie no effect of assessment mode on mental health measures.
